# Single Derivation Fragmented QRS Can Predict Poor Prognosis in Successfully Revascularized Acute STEMI Patients

**DOI:** 10.5935/abc.20170099

**Published:** 2017-09

**Authors:** Zulkif Tanriverdi, Huseyin Dursun, Tugce Colluoglu, Dayimi Kaya

**Affiliations:** 1 Balikligol State Hospital - Clinic of Cardiology, Turkey; 2 Dokuz Eylul University - Faculty of Medicine - Department of Cardiology, Turkey

**Keywords:** Myocardial Infarction/diagnosis, Percutaneous Coronary Intervention, Electrocardiography, Hospital Mortality, Myocardial Revascularization

## Abstract

**Background:**

QRS fragmentation (fQRS) is classically defined as the presence of slurred QRS morphology in at least two contiguous leads, and its prognostic importance has been shown in ST elevation myocardial infarction (STEMI). However, no study has investigated the significance of single lead fQRS (sl-fQRS) in surface electrocardiography (ECG).

**Objectives:**

To evaluate whether sl-fQRS is as valuable as classical fQRS in patients with acute STEMI who had successful revascularization with primary percutaneous coronary intervention (pPCI).

**Methods:**

We included 330 patients with a first STEMI who had been successfully revascularized with pPCI. The patient’s electrocardiography was obtained in the first 48 hours, and the patients were divided into three groups according to the absence of fQRS (no-fQRS); fQRS presence in a single lead (sl-fQRS); and ≥2 leads with fQRS (classical fQRS).

**Results:**

In-hospital mortality was significantly higher both in patients with sl-fQRS and in patients with ≥ 2 leads with fQRS compared to patients with no-fQRS. In ROC curve analysis, ≥ 1 leads with fQRS yielded a sensitivity of 75% and specificity of 57.4% for the prediction of in-hospital mortality. Multivariate analysis showed that sl-fQRS is an independent predictor of in-hospital mortality (OR: 3.989, 95% CI: 1.237-12.869, p = 0.021).

**Conclusions:**

Although the concept of at least two derivations is mentioned for the classical definition of fQRS, our study showed that fQRS in only one lead is also associated with poor outcomes. Therefore, ≥1 leads with fQRS can be useful when describing the patients under high cardiac risk in acute STEMI.

## Introduction

The main therapeutic strategy for acute ST segment elevation myocardial infarction (STEMI) is the rapid restoration of epicardial blood flow in the infarct related artery (IRA). Primary percutaneous coronary intervention (pPCI) is the most effective and recommended therapeutic intervention for the reperfusion strategy.^1,2^ Studies have shown that successful angiographic reperfusion, which is defined as Thrombolysis in Myocardial Infarction (TIMI) 3 flow in IRA, is associated with good outcomes.^3,4^ Nevertheless, despite successful restoration of epicardial blood flow by pPCI, an important proportion of acute STEMI patients still continue to be at substantial risk because some amount of myocardial necrosis is inevitable. Therefore, there is a need for additional prognostic indicators.

The presence of slurred QRS morphology in at least two contiguous leads is accepted as the classical definition of fQRS on the 12-lead electrocardiogram (ECG).^5^ This includes an additional R wave (R’), notching of the R wave, notching of the downstroke or upstroke of the S wave, or more than one R' (fragmentation).^6^ It originates from inhomogeneous ventricular activation due to ischemic and/or injured myocardium and develops mostly within 48 hours during acute myocardial infarction.^5,7^ The clinical significance of fQRS has been investigated in several studies, and the presence of fQRS was found to be associated with increased mortality, myocardial scarring, cardiac arrhythmias, and adverse cardiac events.^8-10^

Although the relationship between the presence of fQRS in at least two contiguous leads and adverse clinical outcomes is well known in patients with acute myocardial infarction,^10^ the importance of fQRS in only one lead (single lead fQRS, sl-fQRS) in acute STEMI patients who underwent a successful pPCI has not been studied yet. The aim of our study is to investigate whether sl-fQRS is of prognostic importance in patients with acute STEMI who achieved TIMI 3 flow by pPCI.

## Methods

### Patient selection

This study was conducted at Dokuz Eylul University Hospital between January 1, 2009, and June 1, 2014. Patients who had been admitted to the coronary intensive care unit with the diagnosis of first acute STEMI and had undergone a successfully pPCI were retrospectively evaluated. Current guidelines were used for the diagnosis of acute STEMI.^2,11^ Patients who were admitted with acute STEMI for the first time and successfully revascularized with pPCI in our clinic were included in this study. Successful revascularization was defined as post PCI TIMI 3 flow in the IRA, with a residual stenosis < 20%, and absence of stent thrombosis, repeat PCI, coronary dissection/rupture, or death. 24 patients with complete bundle branch block, 10 patients with incomplete right bundle branch block and 2 patients with pacemaker rhythm were excluded from the study. Also, patients who were known to have fQRS prior to STEMI, those with QRS duration ≥ 120 milliseconds, previous history of coronary artery bypass surgery, and patients who did not show TIMI 3 flow after pPCI were excluded from the study. As a result, 330 eligible patients were included in this study. The study was approved by the local ethics committee and study protocol complied with the Declaration of Helsinki.

### Electrocardiography

**Figure 1:**
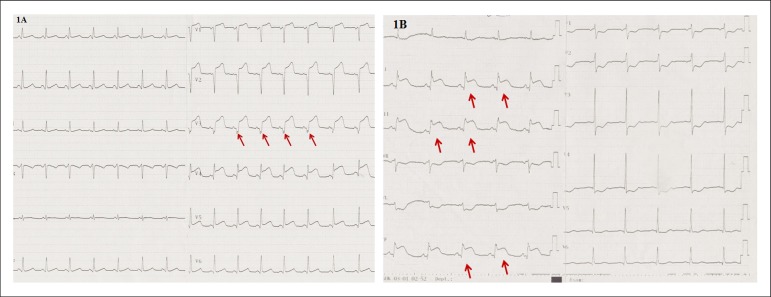
A) ECG example of single lead fQRS in a patient with anterior MI. B) ECG example of ≥ 2 leads with fQRS in a patient with inferior MI.

Twelve-lead ECG was obtained at 25 mm/s paper speed, with a 0.16-100 Hz filter range and 10 mm/mV height from all patients in supine position, on admission, after pPCI, and at the 24^th^ and 48^th^ hours after admission to hospital. Routine ECG analyses were performed with the naked eye and without using any magnification by two independent clinicians. Pre-PCI sum of ST elevations and post-PCI sum of ST elevations were measured, and the percentage of total ST resolution (STR) calculated.^12^ Fragmented QRS was defined by the presence of various RSR’ patterns (QRS duration < 120 ms) with or without Q wave, which include an additional R wave (R’ prime) or notching of the R wave or S wave, or the presence of more than one R' (fragmentation) without typical bundle branch block.^4^ The presence of these criteria in two or more contiguous leads was required for the classical definition of fQRS. However, we also investigated the patients who had the criteria of a single derivation, and we divided the patients into three groups according to the fQRS derivation numbers at 48th hours: absence of fQRS in any lead (no-fQRS), its presence in a single lead (sl-fQRS) (Figure 1A), and its presence in two or more contiguous leads (classical fQRS) (Figure 1B).

### Coronary angiography

Coronary angiography and PCI procedures were performed at the catheterization laboratory through the femoral/radial artery using the standard Judkins technique. Anticoagulant and antiplatelet therapies before PCI were given to all patients according to current guideline.^2^ Angiographic data was assessed by two independent cardiologists. TIMI flow grading system was used to evaluate blood flow in the IRA.^13^ Patients who achieved TIMI 3 flow after pPCI were included in this study. The presence of stenosis ≥ 50% in the left main coronary artery and ≥ 70% in the other major epicardial coronary arteries was considered critical stenosis.

### Statistical analysis

Statistical analysis was performed using SPSS for Windows version 22.0 (SPSS Inc., Chicago, IL, USA). Kolmogorov-Smirnov test was used to determine normality of distribution. Continuous variables were tested for normal distribution using Kolmogorov-Smirnov test. Continuous variables were expressed as the mean ± standard deviation, and categorical variables were expressed as percentages. Continuous variables were compared with the one-way analysis of variance (ANOVA). A posteriori tests were performed after ANOVA to study differences between groups. Categorical variables were compared with chi-square or Fisher’s exact tests. Correlation analysis between continuous variables was done by Pearson’s method. A receiver operating characteristic (ROC) curve was used to determine the best cut-off number of the leads with fQRS in the prediction of in-hospital mortality. Multivariate logistic regression analysis was performed to determine the independent predictors of in-hospital mortality. A P value of < 0.05 was considered to be statistically significant.

## Results

Three hundred-thirty patients who underwent a successful pPCI were included in this study. Baseline characteristics of patients are listed in Table 1.

**Table 1:** Baseline characteristics of patients

	(n = 330)
Age (years)	60.2 ± 13.2
Gender M/F	259/71
Hypertension (%)	151 (45.8)
Diabetes Mellitus (%)	77 (23.3)
Chest pain duration on admission (min.)	169.5 ± 184.3
Door to balloon time (min.)	21.5 ± 4.6
LVEF (%)	40.8 ± 8.7
Maximum CK-MB	145.2 ± 103.3
Maximum Troponin	38.2 ± 23.7
Number of STE derivations	5.0 ± 1.6
Number of STD derivations	3.1 ± 1.6
No leads with fQRS (%)	179 (54.2)
One lead with fQRS (%)	45 (13.6)
≥ 2 leads with fQRS (%)	106 (32.1)
Mean number of leads with fQRS	1.2 ± 1.8
**MI localization**	
Anterior (%)	178 (53.9)
Non-anterior (%)	152 (46.1)
Pre-PCI sum of STE	10.6 ± 7.0
Post-PCI sum of STE	3.7 ± 3.1
STR ratio (%)	65.1 ± 25.0
**Infarct-related artery**	
LAD (%)	178 (53.9)
CX (%)	53 (16.1)
RCA (%)	99 (30)
**Stent type**	
BMS (%)	94 (28.5)
DES (%)	236 (71.5)
Glycoprotein IIb-IIIa inhibitors (%)	29 (8.8)
Number of vessels with critical stenosis	1.8 ± 0.8
Three-vessel disease (%)	80 (24.2)
In-hospital mortality (%)	32 (9.7)

BMS: bare metal stent; CK-MB: creatinine kinase-MB; CX: circumflex artery; DES: drug eluting stent; F: female; fQRS: Fragmented QRS; LAD: left anterior descending artery; LVEF: left ventricular ejection fraction; M, male; MI, myocardial infarction; min, minute; PCI, percutaneous coronary intervention; RCA, right coronary artery; STD, ST depression; STE: ST elevation; STR, ST resolution.

Our study group was divided into three groups according to the number of leads with fQRS: no lead with fQRS; only one lead with fQRS; and ≥ 2 leads with fQRS. The higher number of leads with fQRS on surface ECG was significantly related with lower left ventricular ejection fraction (LVEF) (p < 0.001), lower STR ratio (p < 0.001), higher maximum CK-MB and troponin (p < 0.001 and p< 0.001), higher number of vessels with critical stenosis (p < 0.001), higher frequency of three-vessel disease (p < 0.001), and higher rate of in-hospital mortality (p = 0.002) (Table 2).

**Table 2:** Comparison of clinical, electrocardiographic, and angiographic characteristics of patients according to the number of leads with fQRS

	no-fQRS (n = 179)	sl-fQRS (n = 45)	Classical fQRS (n = 106)	p*
Age (years)	59.7 ± 13.1	57.9 ± 14.3	62.1 ± 12.8	0.149
Gender M/F	140/39	35/10	84/22	0.972
Hypertension (%)	77 (43)	20 (44.4)	54 (50.9)	0.423
Diabetes Mellitus (%)	34 (19)	12 (26.7)	31 (29.2)	0.120
Duration of chest pain on admission (min.)	159.9 ± 174.2	172.7 ± 155.7	184.4 ± 210.7	0.550
Door to balloon time (min.)	21.5 ± 4.7	21.6 ± 5.2	21.4 ± 4.2	0.986
LVEF (%)	44.7 ± 7.5	41.0 ± 8.6	34.2 ± 6.4	< 0.001
Max. CK-MB (ng/ml)	111.1 ± 84.9	122.4 ± 89.9	212.3 ± 105.3	< 0.001
Max. Troponin (ng/ml)	29.2 ± 18.3	38.9 ± 24.0	53.2 ± 24.1	< 0.001
Number of STE derivation	5.1 ± 1.6	4.9 ± 1.8	4.9 ± 1.6	0.785
Number of STD derivation	3.0 ± 1.7	3.0 ± 1.6	3.2 ± 1.6	0.632
Mean number of leads with fQRS	0.0 ± 0.0	1.0 ± 0.0	3.3 ± 1.6	< 0.001
**MI localization (%)**				
Anterior	103 (57.5)	21 (46.7)	54 (50.9)	0.320
Non-Anterior	76 (42.5)	24 (53.3)	52 (49.1)	
STR ratio (%)	74.9 ± 15.5	63.9 ± 28.3	49.1 ± 28.0	< 0.001
**Stent type**				
BMS (%)	59 (33)	12 (26.7)	23 (21.7)	0.121
DES (%)	120 (67)	33 (73.3)	83 (78.3)	
Glycoprotein IIb-IIIa inhibitors (%)	17 (9.5)	4 (8.9)	8 (7.5)	0.854
Number of vessels with critical stenosis (%)	1.5 ± 0.7	1.8 ± 0.8	2.2 ± 0.8	< 0.001
Three-vessel disease (%)	17 (9.5)	11 (24.4)	52 (49.1)	< 0.001
In-hospital mortality (%)	8 (4.5)	6 (13.3)	18 (17)	0.002

BMS: bare metal stent; Classical fQRS, ≥ 2 leads with fQRS; CK-MB: creatinine kinase MB; DES: drug-eluting stent; F: female; fQRS: Fragmented QRS; LVEF: left ventricular ejection fraction; M: male; MI: myocardial infarction; min: minute; QRS; sl-fQRS, Single lead fragmented QRS; STD: ST depression; STE: ST elevation; STR: ST resolution

* ANOVA and Chi-square tests were performed to study differences among the three groups. A posteriori test (Tukey) was performed after ANOVA to study between group differences for no-fQRS vs. sl-fQRS, no-fQRS vs. classical fQRS and sl-fQRS vs. classical fQRS.

**Figure 2:**
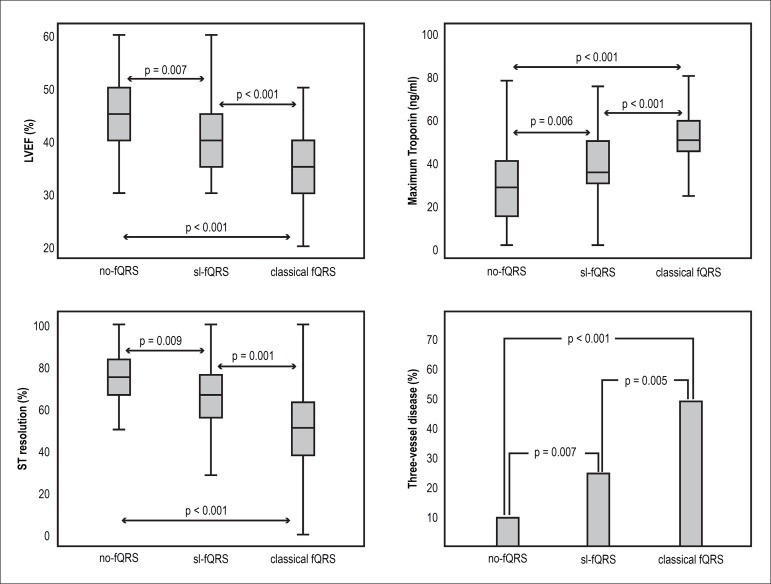
Comparisons among groups in terms of LVEF, maximum troponin, ST resolution, and the frequency of three-vessel disease. LVEF: left ventricular ejection fraction; fQRS: Fragmented QRS.

**Figure 3:**
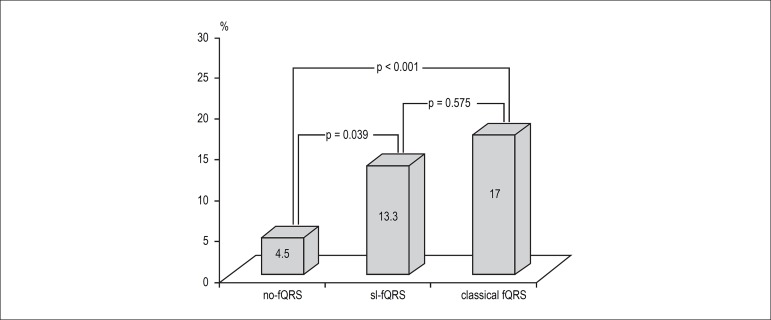
Comparisons among groups in terms of in-hospital mortality.

To better elucidate the importance of sl-fQRS, these patients were compared to those with no-fQRS and those with ≥ 2 leads with fQRS. Patients with sl-fQRS had a lower LVEF (41.0 ± 8.6 vs. 44.7 ± 7.5, p = 0.007), a lower ratio of STR (63.9 ± 28.3 vs. 74.9 ± 15.5, p = 0.009), higher maximum troponin levels (38.9 ± 24.0 vs. 29.2 ± 18.3, p = 0.019), and a higher rate of three-vessel disease (24.4% vs. 9.5%, p = 0.007) than patients with no-fQRS. Similarly, patients with ≥ 2 leads with fQRS also had a lower LVEF, a lower ratio of STR, higher maximum troponin levels, and a higher rate of three-vessel disease than patients with sl-fQRS (Figure 2). Hospital mortality was significantly higher in patients with sl-fQRS compared to patients with no-fQRS (13.3% vs 4.5%, p = 0.039), but it was not different between in patients with sl-fQRS and those with ≥ 2 leads with fQRS (Figure 3).

Correlation analysis showed that as the number of fQRS derivations increased, maximum troponin (r = 0.389, p < 0.001) and the number of vessels with critical stenosis (r = 0.399, p < 0.001) increased significantly; conversely, STR (r = -0.506, p < 0.001) and LVEF (r = -0.520, p < 0.001) decreased significantly.

**Figure 4:**
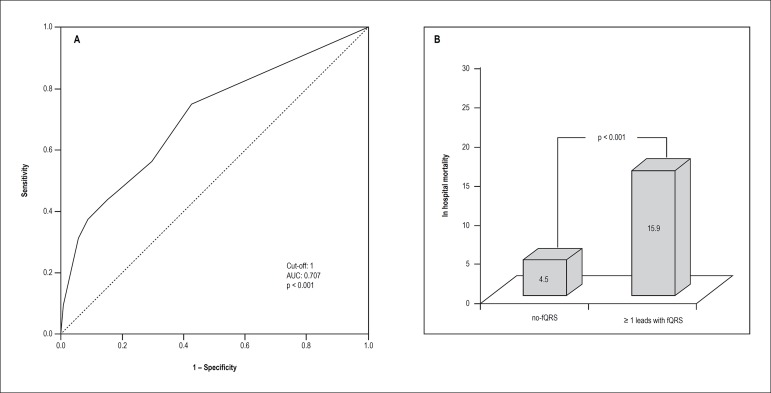
A) ROC curve to determine the best cut-off for number of leads with fQRS in the prediction of in-hospital mortality. B) In-hospital mortality rate in no-fQRS and ≥ 1 leads with fQRS groups.

In ROC curve analysis, ≥ 1 leads with fQRS yielded an area under curve (AUC) value of 0.707 (95% CI: 0.605-0.809, p < 0.001), which demonstrated a sensitivity of 75% and specificity of 57.4% for the prediction of in-hospital mortality (Figure 4A). When our study group was divided into two groups according to this cut-off value, in-hospital mortality was significantly higher for the group with ≥ 1 leads with fQRS (Figure 4B).

Multivariate logistic regression analyses were performed to determine the independent predictors of in-hospital mortality. Sl-fQRS (odds ratio [OR]: 3.989, 95% confidence interval [CI]: 1.237-12.869, p = 0.021), ≥ 2 leads with fQRS (OR: 4.298, 95% CI: 1.739-10.618, p = 0.002), and age (OR: 1.074, 95% CI: 1.039-1.110, p < 0.001) were found to be independent predictors of in-hospital mortality (Table 3). When patients were included as no-fQRS and ≥ 1 leads with fQRS in another model, age (OR: 1.076, 95% CI: 1.041-1.113, < 0.001) and ≥ 1 leads with fQRS (OR: 4.429, 95% CI: 1.851-10.595, p = 0.001) were found to be independent predictors of in-hospital mortality.

**Table 3:** Multivariate logistic regression analysis showing the independent predictors of in hospital mortality

	Predictors	OR	95% CI	p
Model 1*	Age	1.074	1.039-1.110	< 0.001
	sl-fQRS	3.989	1.237-12.869	0.021
	≥ 2 leads with fQRS	4.298	1.739-10.618	0.002
Model 2^†^	Age	1.076	1.041-1.113	< 0.001
	≥ 1 leads with fQRS	4.429	1.851-10.595	0.001

β, β coefficient; CI: confidence interval; OR: odds ratio; SE: Standard error.

* Entered variables: Age, Hypertension, Diabetes mellitus, Duration of chest pain on admission, Door to balloon time, Stent type, CK-MB, Troponin, Number of ST elevated and ST depressed derivations, MI localization, sl-fQRS, ≥ 2 leads with fQRS, Number of affected lesion narrowness >70%, ST segment resolution score.

† Entered variables: Age, Hypertension, Diabetes Mellitus, Duration of chest pain on admission, Door to balloon time, Stent type, CK-MB, Troponin, Number of ST elevated and ST depressed derivations, MI localization, ≥ 1 leads with fQRS, Number of affected lesion narrowness >70%, ST segment resolution score.

## Discussion

The main finding of our study was that in-hospital mortality was significantly higher in patients with sl-fQRS compared to patients with no-fQRS. In addition, LVEF and STR ratio were significantly lower, whereas maximum troponin levels and frequency of three-vessel disease were significantly higher in patients with sl-fQRS than in those with no-fQRS. Our study showed that sl-fQRS and/or ≥ 1 leads with fQRS are independent predictors of in-hospital mortality even if TIMI grade 3 flow is achieved by primary PCI in acute STEMI patients.

Significant QRS fragmentation on surface ECG was defined as the presence of slurred QRS morphology in two or more contiguous leads, and only one lead with fQRS was not accepted as the presence of fQRS.^5^ Therefore, the importance of the presence of fQRS at ≥ 2 lead has usually been investigated in studies, and it has been found to predict poor prognostic events in acute STEMI patients.^14,15^ The importance of fQRS has also been investigated in coronary artery disease and non-ischaemic cardiomyopathy in a previous meta-analysis conducted by Rosengarten et al.^16^ They also used the classical definiton for the presence of fQRS and excluded studies which used an alternative definition for fQRS. They found that fQRS was associated with all-cause mortality and the occurrence of sudden cardiac death. However, we think this classic definition may lead to overlook some patients who actually have high risk. That is because there is no study showing the importance of the presence of fQRS in a single derivation in patients with acute STEMI. To the best of our knowledge, ours is the first study that demonstrated the significance of sl-fQRS in acute STEMI patients who underwent a successful pPCI.

It is known that final TIMI ≤ 2 flow after pPCI is strongly associated with poor outcomes.^3^ Therefore, these patients were not included in our study to avoid the effect of TIMI ≤ 2 flow on mortality. All patients in our study are the patients who underwent a successful pPCI, which means that these patients had lower necrotic myocardium so that angiographic TIMI 3 flow had been achieved. Despite successful revascularization with pPCI, in-hospital mortality rate of our study was 9.7%. This may be due to the small number of patients with respect to the current volume of pPCI and relatively higher rate of anterior MI (53.9%).

Clinical features, duration of chest pain, and MI localization were similar in the three groups. However, we found that in-hospital mortality was significantly higher in patients with sl-fQRS compared to patients with no-fQRS, and ≥ 1 leads with fQRS yielded a sensitivity of 75% and specificity of 57.4% for the prediction of in-hospital mortality. In addition, sl-fQRS was independent predictor of in-hospital mortality. As we showed that sl-fQRS was an independent predictor of in-hospital mortality, we constructed a new regression model, in which patients were included as no-fQRS and ≥ 1 leads with fQRS. We found that ≥ 1 lead with fQRS was independent predictor of mortality. More importantly, the odds ratio of ≥ 1 leads with fQRS (4.429) was higher than odds ratio of ≥ 2 leads with fQRS (4.298). Although previous studies showed the presence of fQRS in two or more contiguous leads was associated with increased in-hospital mortality,^10^ this is first study demonstrating the relationship between sl-fQRS, ≥ 1 leads with fQRS and in-hospital mortality. Celikyurt et al.,^17^ found that the number of leads with fQRS was the only predictor of response to cardiac resynchronization therapy, and the best cut-off number of leads with fQRS to distinguish between responder and non-responder patients was one. These findings suggest that the presence of fQRS even in just one lead can be of prognostic significance. Furthermore, in-hospital mortality was similar between patients with sl-fQRS and those with ≥ 2 leads with fQRS in our study. This also suggests that sl-fQRS could be as significant a finding as classical fQRS in patients with acute STEMI.

Only one previous case report has assessed the association between fQRS in just one lead and myocardial scar.^18^ In this case presentation, fQRS in lead V3 alone, without other electrocardiographic abnormalities, may be because myocardial infarction was limited to a narrow area of the left ventricular apex. However, there is no other information in literature about the importance of the fQRS in one lead alone in patients with acute STEMI. In this study, we detected that patients with sl-fQRS had a lower LVEF, higher maximum troponin levels, and a higher rate of three-vessel disease than patients with no-fQRS. Therefore, it can be suggested that the presence of fQRS even in one lead is also associated with the necrosis of certain amount of myocardial tissue. We think further studies with larger sample sizes are needed to better clarify the mechanism and clinical significance of fQRS in one lead alone.

It is known that the presence of fQRS is associated with lower STR in acute STEMI patients.^19,20^ Coronary artery patency has been assessed with TIMI flow in clinical practices, but recent studies have shown that STR is a stronger marker than angiographic TIMI flow to evaluate tissue reperfusion and predict cardiac outcomes.^21,22^ Although in this study TIMI 3 flow was provided in all patients after pPCI, we found that STR is lower in patients with sl-fQRS when compared to in patients with no lead with fQRS. Accordingly, we can conclude that acute STEMI patients who have only one lead with fQRS will also show poor reperfusion at the cellular level even if TIMI grade 3 flow is achieved by primary PCI.

Fragmented QRS is a novel ECG parameter that is used quite often in daily practice and that is gaining importance.^23^ However, the number of fQRS derivations has recently attracted a greater interest. Even though there is no study showing the importance of the presence of fQRS in a single lead, the clinical significance of QRS distortion in only one lead, which is another important ECG finding in acute STEMI,^24^ was investigated in a recently published study.^25^ Similar to our study, this study first demonstrated that QRS distortion in only one lead was associated with larger infarct size. Based on these results, we suggest that the presence of fQRS in a single derivation also has prognostic importance. This cut-off number of leads with fQRS will need to be validated in larger prospective studies.

One of the major limitations of this study is that we did not use the TIMI myocardial perfusion degree or myocardial blushing grade, which are the other parameters of angiographic reperfusion. These parameters could have provided additional benefits to our study. In addition, the findings of this study cannot be generalized to all acute STEMI patients since patients who underwent thrombolytic therapy, those with QRS duration ≥ 120 milliseconds, and those for whom it was not the first acute STEMI, were not included to the study.

## Conclusion

The concept of at least two derivations is mentioned for the classical definition of fQRS, and only one lead with fQRS has not been accepted for the presence of fQRS. However, we showed for the first time that sl-fQRS is associated with greater extent of necrotic myocardium, increased in-hospital mortality and higher risk. Therefore, instead of the concept of at least two derivations, the presence of fQRS in only one lead and/or ≥1 leads with fQRS may also be enough when describing the patients under high cardiac risk. Further studies are needed to understand the importance of sl-fQRS.
